# Validation of 'Quick Sequential Organ Failure Assessment Score' as a Screening Tool for Early Identification of Sepsis Patients in the Emergency Department

**DOI:** 10.7759/cureus.39251

**Published:** 2023-05-19

**Authors:** Zuhaib Ahmed Wani, Khushboo Gulzar, Hilal Yatoo, Tamorish Kole

**Affiliations:** 1 Emergency, Sher-i-Kashmir Institute of Medical Sciences Medical College & Hospital, Srinagar, IND; 2 Obstetrics and Gynaecology, Directorate of Health Services Jammu & Kashmir, Srinagar, IND; 3 Emergency, Epsom and St Helier University Hospitals, Sutton, GBR; 4 Emergency Medicine, Medhavi Skillversity, Gurugram, IND

**Keywords:** emergency department, new score, qsofa, septic shock, sepsis

## Abstract

Sepsis and septic shock are major healthcare problems, affecting millions of people around the world each year. The speed and appropriateness of therapy administered in the initial hours of treatment are likely to influence the outcome.

We conducted a study to validate the clinical assessment score named 'quick sequential organ failure assessment' (qSOFA) score for use in the early identification of sepsis patients in the emergency department. Our primary objective was to see the sensitivity and specificity of the qSOFA-score for diagnosing sepsis in the emergency department and our secondary objective was to compare the sensitivity of the qSOFA score with the National Early Warning (NEW) score in patients with sepsis.

A prospective observational study was conducted at Max Super Speciality Hospital Saket, New Delhi, from July 2016 to January 2017. Adult patients presenting to the emergency department with clinical signs and symptoms suggestive of infection were enrolled as per the eligibility criteria and divided into two groups on the basis of their qSOFA score at presentation.

Out of 120 patients who had a positive qSOFA score 30 were subsequently confirmed as having sepsis whereas in qSOFA negative group 14 patients were subsequently diagnosed as having sepsis. This leads to the fact that although the test has near-acceptable specificity, the sensitivity is quite low. Calculations of the secondary outcome, that is 28-day mortality, revealed that 17 patients out of 120 who had a positive qSOFA score died within 28 days of first presentation whereas in the control group, nine patients had died. This means it successfully predicted mortality in only 17 patients and failed to predict mortality in nine patients out of 26 patients that died. The p-value is 0.097 which indicates both poor sensitivity as well as specificity for predicting mortality. We also compared qSOFA with the NEW score and found the latter to have a better sensitivity for detecting sepsis.

This study shows that the qSOFA score, which has been specifically designed for early detection of sepsis patients in the emergency department or a pre-hospital setting in whom infection is suspected on a clinical basis, does not seem to be a good screening tool for early detection of sepsis patients in the emergency department.

## Introduction

Sepsis is a life-threatening condition that arises when the body's response to an infection injures its own tissues and organs [1]. As per the 1991 ACCM (American College of Critical Care Medicine) and SCCM (Society of Critical Care Medicine) definitions, later updated in 2008 & 2012, sepsis is defined as the presence of infection together with systemic manifestations of infection [2].

Systemic inflammatory response syndrome (SIRS) is the presence of two or more of the following: abnormal body temperature, heart rate, respiratory rate, blood gas, and white blood cell count [3]. Sepsis is also defined as SIRS in response to an infectious process [4]. Septic shock is sepsis plus persistently low blood pressure despite the administration of intravenous fluids [5].

Sepsis and septic shock are major healthcare problems, affecting millions of people around the world each year, killing nearly one in four affected individuals, and the incidence increasing [6]. Early diagnosis is necessary to properly manage sepsis, as an initiation of early goal-directed therapy is key to reducing mortality [5].

In 2016 an updated definition of sepsis was proposed by the European Society of Intensive Care Medicine and the SCCM (Sepsis-3 task force) [1], according to which sepsis should be defined as “Life-threatening organ dysfunction caused by a dysregulated host response to infection”. For clinical operationalization, organ dysfunction can be represented by an increase in the Sequential Organ Failure Assessment (SOFA) score of 2 points or more [7].

In the process of development of these new definitions, a new scoring system named qSOFA (quick sequential organ failure assessment score) was formulated through retrospective data analysis. A clinical model developed with multivariable logistic regression identified that any two of the three clinical variables viz Altered Mental Status, Systolic BP & Respiratory Rate offered a predictive validity similar to that of full SOFA score outside the ICU [1]. qSOFA provides simple bedside criteria to identify adult patients with ‘suspected infection’ who are likely to have sepsis.

The NEW (National Early Warning) Score comparatively consists of a larger number of variables. It is being used in emergency rooms across Europe to identify critically ill patients and is not used specifically for sepsis. The aggregate NEW score determines the ‘urgency of response’ and is not used as a diagnostic or prognostic indicator. NEW score update to NEWS2 was proposed in the year 2017 wherein a scale-two was suggested for patients whose SpO2 falls between 88-92%, this was mainly done to help better evaluation of chronic obstructive pulmonary disease (COPD) patients for whom this range of SpO2 is recommended [8]. NEWS2 is still pending a review (expected in the current year 2023).

While no single individual can be termed as the inventor or father of sepsis, Roger Bone (1970-1990) spearheaded an attempt to bring organisation to the literature and clinical trials concerning sepsis [5].

The guidelines concerning the diagnosis of sepsis, severe sepsis and septic shock were revised in 2008 and again in 2012 but with little change to the definitions or the diagnostic criteria. The year 2012 saw the introduction of the ‘surviving sepsis campaign’ and the concept of three- and six-hour bundles [5].

The Third International Consensus Definitions Task Force (February 2016) defined sepsis as “life-threatening organ dysfunction due to a dysregulated host response to infection”. The objective was to evaluate the validity of clinical criteria to identify patients with suspected infection who are at risk of sepsis [1]. There being no universally accepted medical definition of infection, clinicians world over for long have been using the presence of specific signs and symptoms, examination or test results as indicators of infection. They made a retrospective data analysis from the health record system of about 1.3 million patients in Pennsylvania, USA. A primary cohort of 148,907 patients with suspected infection was taken and divided into derivation and validation cohorts. SOFA score, SIRS criteria, Logistic Organ Dysfunction System (LODS) score, and the new model named qSOFA score were measured and compared for in-hospital mortality and ICU length of stay ≥3 days. Results were expressed as changes in outcome over deciles of baseline risk of death and area under the receiver operating characteristic curve (AUROC).

Among non-ICU encounters in the validation cohort (n = 66,522 with suspected infection, of whom 1886 died), qSOFA had predictive validity (AUROC = 0.81; 95% CI, 0.80-0.82) that was greater than SOFA (AUROC = 0.79; 95% CI, 0.78-0.80; P < .001) and SIRS (AUROC = 0.76; 95% CI, 0.75-0.77; P < .001) [9]. The predictive validity for in-hospital mortality of qSOFA was statistically greater than SOFA and SIRS, supporting its use as a prompt to consider possible sepsis [10].

There was a study of 30,677 patients in the emergency department and ward at the University of Chicago. Electronic records were retrospectively analysed to calculate SIRS and qSOFA scores. These scores were compared to a primary outcome of in-hospital mortality and a combined outcome of mortality or ICU admission. In predicting mortality or ICU transfer (arguably the most relevant outcome), qSOFA and SIRS were similar [11].

In July 2016, Vincent et al. published an article in the journal ‘Critical Care’ wherein the author raised the following points: That SIRS criteria have a low specificity for sepsis, which often leads to overdiagnosis and undue ICU admissions; that qSOFA is meant to be used to raise suspicion of sepsis and prompt further action. It is not a replacement for SIRS and is not part of the definition of sepsis [10].

Recently, it has been suggested that qSOFA be used in conjunction with POC (point of care) serum prolactin results, labelled pqSOFA+ for serum prolactin > 0.25 ng/ml and pqSOFA- for levels < 0.25 ng/ml [12].

## Materials and methods

This was a prospective observational study that was conducted in the department of emergency medicine Max Super Speciality Hospital Saket, New Delhi, from July 2016 to January 2017. The study included patients meeting the inclusion and exclusion criteria, that is, all consecutive patients >18 years of age both male as well as female presenting to the emergency room who are clinically suspected of having an infection. Patients with a qSOFA score of ≥ 2 were included in the case group (n = 120) whereas patients with a qSOFA score of < 2 were taken as controls (n = 120). All referrals or shifts from other hospitals and pregnant patients were excluded from the study. The primary outcome was a diagnosis of sepsis within 24 hours of presentation to the Emergency Department. Whereas the secondary outcome was the 28 days mortality (discharged patients were followed telephonically).

In all the study patients, initial triaging was done by the on-duty triage nurse who recorded the patient’s vitals which included heart rate (HR), respiratory rate (RR), blood pressure (BP), blood gas oxygen saturation (SpO2), temp., and mental status. The patients were subsequently seen by the on-duty Resident doctor who as per protocol took their clinical history and did the requisite physical examination. Patients in whom the initial evaluation led to the suspicion of infection, inclusion and exclusion criteria were applied. Patients who met the criteria were briefed about the study and a written consent was taken from the patient/caregiver after which qSOFA and NEW scores were calculated and filled in the proforma. The patients with a positive qSOFA score (Score ≥ 2) were labelled as cases and those with a negative score (score ≤ 1) as controls. Investigations and treatment were done as per the standard hospital protocol.

The patients were followed till the end of 24 hours, one week and 28 days from the time of presentation through direct contact within the hospital and through the Computerised Patient Record System (CPRS). Patients already discharged from the hospital were followed telephonically.

## Results

qSOFA-positive patients were labelled as Group A whereas qSOFA-negative patients were labelled as Group B. There was no significant difference in the age distribution between the two groups - Group A 52.88 ± 21.12, Group B 49.45 ± 23.54 (P-value 0.249). Though both the groups included a larger male population the difference was small and not significant statistically - Group A had 53.3% males and 46.7% females whereas Group B had 57.5% males and 42.5% females.

Sensitivity, specificity, positive and negative predictive values of qSOFA for sepsis were calculated using MedCal V.12.4.0.0 (MariaKerke, Belgium). The area under the receiver operating characteristic (AUROC) curve was calculated using IBM SPSS Statistics V.20.0.0 (IBM Corp., Armonk, NY, USA). The Chi-square test was used to compare the qSOFA score with the NEW score.

Out of 120 patients who had a positive qSOFA score (Group A), 30 were subsequently confirmed as having sepsis whereas in qSOFA negative group (Group B) 14 patients were subsequently diagnosed as having sepsis (Table 1). This leads to the fact that although the test has near-acceptable specificity, the sensitivity is quite low, having missed 11.7% cases of sepsis is significant.

**Table 1:** Primary outcome The final diagnosis of sepsis was made at the end of 24 hours after the presentation when the patients were reviewed and any change in their full-SOFA scores were noted. An increase of the total SOFA score by two or more points was considered diagnostic of sepsis (as per sepsis-3 guidelines).

Primary Outcome	Group A (qSOFA+)		Group B (qSOFA-)		P-Value
	Frequency	%	Frequency	%	
No Sepsis	90	75.0%	106	88.3%	0.008
Sepsis	30	25.0%	14	11.7%	
Total	120	100%	120	100%	

Calculations of the secondary outcome, that is 28-day mortality (Table 2), revealed that 17 patients out of 120 who had a positive qSOFA score died within 28 days of 1st presentation whereas in the control group nine patients had died. This means it successfully predicted mortality in only 17 patients and failed to predict mortality in nine patients out of a total of 26 deaths. The p-value is 0.097 which indicates both poor sensitivity as well as specificity for predicting mortality.

**Table 2:** Secondary outcome

Secondary outcome	Group A (qSOFA+)		Group B (qSOFA-)		P-value
	Frequency	Percentage	Frequency	Percentage	
Died	17	14.2%	9	7.5%	0.097
Improved	103	85.8%	111	92.5%	
Total	120	100%	120	100%	

The distribution of cases according to the aggregate qSOFA score reveals clustering at score 2 (Table 3) which is the cut-off for labelling the patients as positive (suspected sepsis). This may indicate the need for revision of total number of points assigned to the different parameters within the score.

**Table 3:** Relative distribution of cases and controls at the qSOFA score values. Clustering can be noted at an aggregate score of 2 for the qSOFA positive group.

qSOFA Score	Group A	Group B
0	0.0%	46.7%
1	0.0%	53.3%
2	84.2%	0.0%
3	15.8%	0.0%

The average blood pressure is 99.32 mmHg in Group A and 113.9 mmHg in Group B (Table 4) which means that only a slight decrease in systolic BP below the threshold is noticeable in group A and as such a large number of patients who therefore are just borderline hypotensive end up being qSOFA+. The threshold (systolic BP < 100) thus may need to be revised to increase the specificity of the test. It can be noticed that the difference in mean respiratory rate between the two groups is significant and this parameter is probably a major contributor to the specificity of the test.

**Table 4:** NEW score values for Group A and Group B

	Group A	Group B
Heart Rate	101.88	100.83
Resp. Rate	23.5	18.96
Systolic Bp	99.32	113.9
Diastolic Bp	64.3	72.87
Temp.	37.51	37.72
SPO2 %	94.93	95.43

Table 5 shows that there is little difference in the final outcome in patients who were ultimately diagnosed as having sepsis irrespective of whether they scored positive or negative for qSOFA at the initial presentation, which means that the score is not a good predictor of mortality.

**Table 5:** Mortality (at 28 days) in patients who were later confirmed of having sepsis. Final outcome in patients who were ultimately diagnosed as having sepsis (via full SOFA score) irrespective of whether they scored positive or negative for qSOFA at initial presentation.

Sepsis Diagnosed	qSOFA+	qSOFA-
Died	43.3%	50.0%
Improved	56.7%	50.0%

On the other hand, the average NEW score in Group A was 7.22, whereas it was 4.42 in Group B (Table 6). The NEW score (when we compared medium and high scores) is more sensitive and specific for sepsis as compared to the qSOFA score (Table 7). In patients who were later diagnosed as having sepsis the mean NEW score was 11.27 for the qSOFA+ group and 8.86 for the qSOFA- group, whereas the average NEW score for patients who had sepsis was 10.5. This means that if the threshold for a ‘high’ NEWS score is revised (specifically for sepsis) better specificity can be achieved (Table 8).

**Table 6:** Mean values of NEW score for qSOFA positive and negative patients respectively. qSOFA positive roughly corresponds to an average NEW score of 7.22 or more.

	Group A	Group B
Mean value of News Score	7.22	4.42

**Table 7:** NEW score values for patients at final diagnosis (non-sepsis vs sepsis groups)

NEWS score	Non-Sepsis		Sepsis		P-Value
	Frequency	%	Frequency	%	
Low	98	50.0%	1	2.3%	<0.001
Medium	53	27.0%	3	6.8%	
High	45	23.0%	40	90.9%	
Total	196	100.0%	44	100.0%	

**Table 8:** Comparison of qSOFA and NEW score results for sepsis patients. For patients later 'confirmed of having sepsis' the mean NEW score for group A was 11.27, for group B was 8.86 and the average score was 10.5.

qSOFA score	Sepsis	Percentage	Mean NEW Score	SD
Positive	30	68.2%	11.27	3.778
Negative	14	31.8%	8.86	1.292
Total	44	100%	10.5	3.379

**Figure 1:**
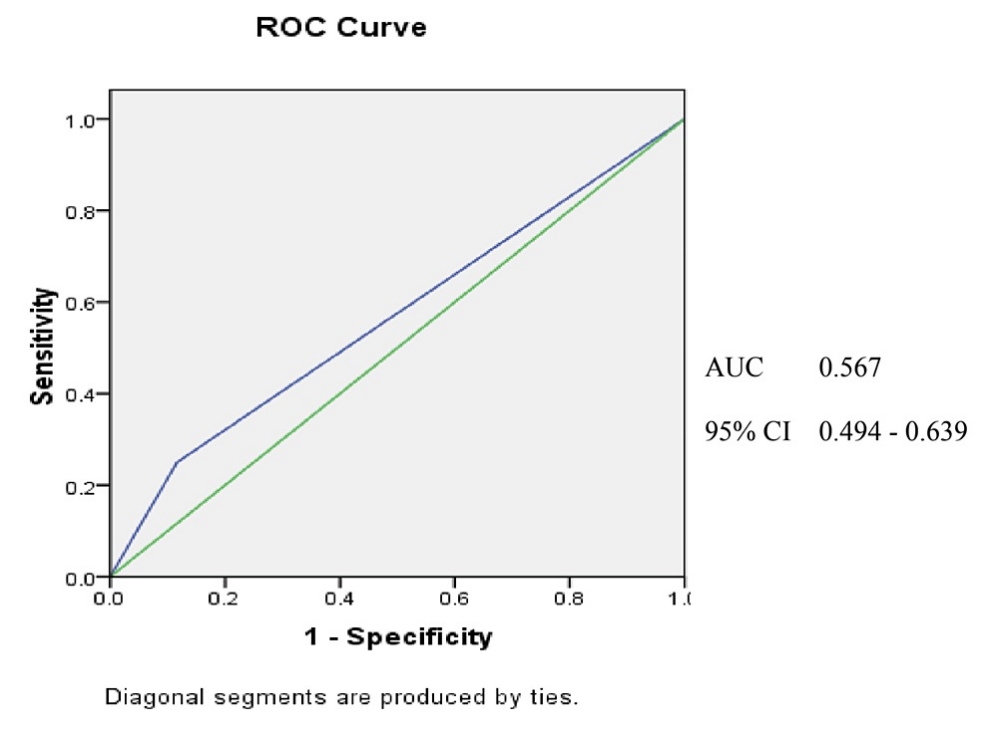
AUROC of qSOFA for sepsis The AUROC of qSOFA for sepsis is 0.567 (for a confidence interval of 95%). The sensitivity of qSOFA for sepsis is 68.18% whereas its specificity is 54.08%. The PPV is 25% and the NPV is 88.3%.

The AUROC curve of qSOFA (Figure 1) for sepsis, the area under the curve is 0.567 (for a confidence interval of 95%) which is quite low and indicates that the test cannot be relied upon for clinical use. The sensitivity of qSOFA for sepsis is 68.18% whereas its specificity is 54.08%. The positive predictive value (PPV) is 25% and the negative predictive value (NPV) is 88.3%.

## Discussion

This study shows that the qSOFA score, which has been specifically designed for the early detection of sepsis patients in the emergency department or a pre-hospital setting among whom infection is suspected on clinical basis, does not seem to be a good screening tool. Though qSOFA has some distinct advantages as a score viz simplicity, minimum constituent variables, minimum requirement for healthcare equipment, minimal requirement of calculations, minimal clinical skill needed for taking the score, and easy reproducibility. Our results are contrary to what the parent study revealed and well consistent with another subsequent study done by Churpek et al. [11].

Our study reveals that qSOFA lacks the fundamental qualities of a good screening tool since the sensitivity and the specificity are both below the minimum acceptable standards. The use of only three parameters in calculating the score though convenient creates a susceptibility for bias, for example, AMS (altered mental status) and tachypnoea can be a presenting feature in many neurological diseases of non-infective etiology but even a slight suspicion of infection in such case will give them a positive qSOFA score and the patients will be labelled as suspected sepsis unless the clinician decides otherwise.

A non-infective condition can present with a clinical suspicion of infection, for example, acute exacerbation of COPD with CO2 narcosis, dysuria in women of reproductive age group, chronic allergic cough, and many more; a patient of acute heart failure, or pulmonary thromboembolism or an unsuspected case of poisoning may present with hypotension, tachypnoea and may even have an altered mental status. A simple case of ruptured ectopic pregnancy can present with abdominal pain, hypotension and tachypnoea. In such situations, a positive qSOFA score will easily tilt the balance in favor of ‘suspected sepsis’, which at times could prove disastrous. This also risks delay in identification of the actual cause of the illness. Thus, we can say that lack of sensitivity and specificity are the major shortcomings of qSOFA score which completely overwhelm its advantages stated earlier. Thus, using qSOFA as a screening tool can at times mislead the caregiver and adversely affect patient management, becoming a cause of distress for the patients and their families. Though it may not seem material individually but will surely be an enormous burden on the healthcare sector as a whole, this is especially true of developing nations wherein healthcare is largely a public sector enterprise and resources most of the times are limited.

Both qSOFA and NEWS though not designed to replace SIRS have one distinct advantage over SIRS, neither of the two scores needs POC (point of care) testing for calculation of the overall score and therefore can be seen as pure screening tools and not as diagnostic ones like SIRS. This also means that if used in conjunction with SIRS right from the beginning may lead to better results.

There is no provision in either score for including a more ‘infection relevant’ parameter like presence of fever, chills, malaise, sore throat, diarrhea, rash, cough, dysuria, etc. Inclusion of such a parameter with due weightage could have made the qSOFA score more sensitive as well as specific but instead, the proponents preferred to identify these important signs and symptoms as ‘clinically suspected infection’ which is a grey area. This can be a cause of ambiguity especially when there are limitations to the workup of these points, e.g., a pre-hospital setting, score being used by triage nurses and paramedics, and patient condition precluding a proper history or examination.

NEW score has better overall results and seems to fulfil the basic requirement of a good screening tool viz high sensitivity. It may therefore only be used as an initial triaging tool in the emergency room in the scenario of clinical suspicion of infection aided by POC tests like serum prolactin, blood lactate and pH levels till time better screening tools for sepsis can be developed.

With the above observations in mind, it seems prudent that a better score can probably be developed by using a combination of parameters from qSOFA, NEWS and SIRS and/or infection-specific variables be added such that better sensitivity and specificity for sepsis is achieved. It could also be a two-step score wherein the first phase would only include clinical variables and the second phase includes any change of the variable and POC tests.

## Conclusions

Considering the global burden of sepsis on the human population it is required that a sensitive and relatively specific screening tool be formulated for early identification of patients. qSOFA though not an ideal tool is a step in the right direction. Its simplicity and ease of operation is unmatched but available data has failed to prove its accuracy and reliability. We therefore recommend that qSOFA score in the current form should not be used for labelling patients as suspected cases of sepsis. Instead, the NEW score may be used, or its interpretation be modified such that an aggregate score of 10 and above in the setting of clinically suspected infection be taken as an indicator of sepsis and further evaluation or interventions done accordingly, or the recently suggested modification that is ‘pqSOFA score’ (involves POC serum prolactin test) be used wherever feasible. The current scenario mandates further studies in this field until better scores are developed.

## References

[1] Singer M, Deutschman CS, Seymour CW (2016). The third international consensus definitions for sepsis and septic shock (Sepsis-3). JAMA.

[2] Bone RC, Balk RA, Cerra FB (1992). Definitions for sepsis and organ failure and guidelines for the use of innovative therapies in sepsis. Chest.

[3] (1992). American College of Chest Physicians/Society of Critical Care Medicine Consensus Conference: definitions for sepsis and organ failure and guidelines for the use of innovative therapies in sepsis. Crit Care Med.

[4] Levy MM, Fink MP, Marshall JC (2003). 2001 SCCM/ESICM/ACCP/ATS/SIS International Sepsis Definitions Conference. Crit Care Med.

[5] Dellinger RP, Levy MM, Rhodes A (2013). Surviving sepsis campaign: international guidelines for management of severe sepsis and septic shock: 2012. Crit Care Med.

[6] Jonathan J (2011). Septic shock. Tintinalli's Emergency Medicine: A Comprehensive Study Guide (7th edition).

[7] Vincent JL, Moreno R, Takala J (1996). The SOFA (Sepsis-related Organ Failure Assessment) score to describe organ dysfunction/failure. Intensive Care Med.

[8] Smith GB, Redfern OC, Pimentel MA (2019). The National Early Warning Score 2 (NEWS2). Clin Med (Lond).

[9] Shankar-Hari M, Phillips GS, Levy ML (2016). Developing a new definition and assessing new clinical criteria for septic shock: for the third international consensus definitions for sepsis and septic shock (Sepsis-3). JAMA.

[10] Vincent JL, Martin GS, Levy MM (2016). qSOFA does not replace SIRS in the definition of sepsis. Crit Care (London).

[11] Churpek MM, Snyder A, Han X, Sokol S, Pettit N, Howell MD, Edelson DP (2017). Quick sepsis-related organ failure assessment, systemic inflammatory response syndrome, and early warning scores for detecting clinical deterioration in infected patients outside the intensive care unit. Am J Respir Crit Care Med.

[12] Yu H, Nie L, Liu A (2019). Combining procalcitonin with the qSOFA and sepsis mortality prediction. Medicine (Baltimore).

